# Supplementary material for: MetaRibo-Seq measures translation in microbiomes
Source: Nat Commun. 2020 Jun 29;11:3268. doi: 10.1038/s41467-020-17081-z (PMC7324362; doi:10.1038/s41467-020-17081-z)
Supplement: Supplementary file 10 — Supplementary Data 7 [file 41467_2020_17081_MOESM10_ESM.zip › File2/Confidence_High_Taxonomy/320209_out.krona.html]

Javascript must be enabled to view this page.

members
magnitude
magnitudeUnassigned
count
unassigned
taxon
rank

320209\_out

18

2
superkingdom
18

1224
phylum
18

18
1236
class

91347
order
18

family
3

SRS019068\_contig\_number\_35971SRS063127\_contig\_number\_contig-100\_16376.16377SRS143014\_contig\_number\_contig-100\_290.291
18
543


SRS140492\_contig\_number\_32284
15
genus
1
561

562
species
14

SRS012273\_contig\_number\_48301SRS017191\_contig\_number\_16095SRS017521\_contig\_number\_32899SRS049402\_contig\_number\_contig-100\_408.114023SRS064276\_contig\_number\_21435SRS075947\_contig\_number\_27618SRS077127\_contig\_number\_5030SRS098881\_contig\_number\_13439SRS105082\_contig\_number\_3204SRS142781\_contig\_number\_1163SRS144362\_contig\_number\_31937SRS146888\_contig\_number\_15295SRS147022\_contig\_number\_13872SRS147151\_contig\_number\_1965
